# A Novel In-House Enzyme-Linked Immunosorbent Assay for Genotype 3 Hepatitis E Virus Reveals High Seroprevalence in Blood Donors in Northern Argentina

**DOI:** 10.3389/fmicb.2019.02481

**Published:** 2019-11-01

**Authors:** Lorena Paola Arce, Melisa Florencia Müller, Alfredo Martinez, Armin Baiker, Gabriela Marranzino, Felicitas Agote, Maria Guadalupe Vizoso-Pinto

**Affiliations:** ^1^Infection Biology Laboratory, Instituto de Investigaciones en Medicina Molecular y Celular Aplicada (IMMCA), SIPROSA, CONICET, UNT, San Miguel de Tucumán, Argentina; ^2^Laboratorio de Ciencias Básicas and Or. Genética, Facultad de Medicina de la Universidad Nacional de Tucumán, Tucumán, Argentina; ^3^Centro de Educación Médica e Investigaciones Clínicas (CEMIC), Buenos Aires, Argentina; ^4^Bavarian Health and Food Safety Authority (LGL), Oberschleissheim, Germany; ^5^Banco Central de Sangre de Tucumán “Dr. César Guerra,” PRIS–Si.PRO.SA, San Miguel de Tucumán, Argentina

**Keywords:** hepatitis E virus, enzyme-linked immunosorbent assay, seroprevalence, blood donors, recombinant protein

## Abstract

The Hepatitis E virus (HEV) is an emergent virus that causes acute hepatitis in immunocompetent hosts and chronic hepatitis in immunocompromised hosts. In Latin America, the main circulating genotype HEV-3 is usually of zoonotic origin. Diagnosis and seroprevalence studies mainly rely on the detection of specific antibodies. There are scarce data on the seroprevalence of HEV infection in Latin America mainly due to the lack of awareness of HEV circulation. Furthermore, in some countries, like Argentina, HEV testing is not included in routine assays. In order to provide tools to deepen the knowledge on HEV epidemiology in South America, we designed a new in-house ELISA based on the native recombinant protein ORF2 aa112–608 and demonstrated its potential for detecting anti-HEV immunoglobulin G (IgG) in human serum samples. The following conditions were determined: an optimal antigen concentration of 0.25 μg/ml, a serum dilution of 1:80, gelatin as a blocking agent, and a secondary antibody dilution of 1:2000. A relative sensitivity of 93.33% (95% CI: 77.9–99.2%) and a relative specificity of 99.4% (95% CI: 96.7–100%) were determined using a panel of previously characterized sera and a gold standard (HEV IgG ELISA, DIA.PRO, Italy). Further, we obtained a very good agreement (*κ* index = 0.94, 95% CI: 0.87–1.00) with the gold standard. We screened 813 blood donor samples with this newly developed ELISA and found a seroprevalence of 9.23% (95% confidence interval, 7.33–11.43%). We show for the first time evidence of past HEV infection in Tucuman, the most populated city in northern Argentina. We expect that this study will raise the interest of health decision makers who should intercede to include indirect testing of HEV in regular diagnostic protocols. In conclusion, the in-house ELISA developed in this work shows a very good agreement with an already licensed commercial HEV IgG ELISA (DIA.PRO, ITALY), which can be used as an epidemiologic tool for HEV surveillance.

## Introduction

The Hepatitis E virus (HEV) is an emergent virus that is causing hepatitis worldwide. The clinical presentation of HEV infection varies from mild and self-limiting to severe cases with typical features of hepatitis: malaise, abdominal, muscle and joint pain, anorexia, and jaundice (Purcell and Emerson, 2008; Lewis et al., 2010), which can end up as fulminant hepatitis. Furthermore, chronic hepatitis has recently been reported in patients with immune disorders and that is hepatically compromised (Bricks et al., 2019; Yang et al., 2019). For unclear reasons, the incidence of fulminant hepatitis in pregnant women is up to 20% (Purcell and Emerson, 2008; Lewis et al., 2010). Several extra-hepatic manifestations such as arthralgia, Guillain-Barré syndrome, meningitis, and others have been attributed to HEV infection (Krain et al., 2014; Dalton et al., 2016; Pasha et al., 2018). These aspects of the disease underline the need to further investigate HEV, improve diagnostics, and increase the awareness of its circulation.

HEV-3 circulates in several countries in Europe and America and is transmitted zoonotically mainly by wild boars, domestic pigs, and deers. HEV genomic RNA and replication intermediates have also been detected in donkeys, goats, horses, macaques, mongoose, rabbits, rats, and sheep (Pavio et al., 2010; Kenney, 2019). The main infection mechanism seems to be the consumption of contaminated raw or undercooked meat (Cai et al., 2017; Cook et al., 2017) or contact with infected animals (Vonesch et al., 2019). Certain concern has arisen regarding the need to control blood transfusion units for the presence of HEV nucleic acids (Kraef et al., 2018; Harvala et al., 2019; Rivero-Juarez et al., 2019). Still, there is no clear consensus on whether HEV detection should be included in routine blood product screenings.

Hepatitis E can be diagnosed by the detection of viral RNA in blood and feces by end-point RT-PCR or qRT-PCR, but more accessible diagnostic assays are ELISA or immunoblotting to detect specific antibodies. Direct-to-consumer-testing laboratories in developing countries have limited access to HEV diagnostic tests. In South America, there is scarce data on the epidemiology of HEV. HEV-1 has been detected only in Venezuela and Uruguay in isolated cases (Mirazo et al., 2014). In the rest of the continent, HEV-3 has been isolated from patients and environmental samples; the most frequent subtypes reported were: −3a, −3b, −3c and −3i, which were related to European, American, and Japanese strains (Pisano et al., 2018b). In the 1990s, a 1.8% seroprevalence of anti-HEV antibodies was found in blood donors (*n* = 2,157 samples) in Buenos Aires (Rey et al., 1997). The next epidemiological study looking for specific anti-HEV antibodies in blood donors was carried out also in Buenos Aires in 2012 by Munne et al. who found a seroprevalence of 10.6% in 123 adults voluntarily screened on the World Hepatitis Day (Munne et al., 2014).

Further evidence of past infections was found in epidemiological studies of specific patient groups such as immunocompromised individuals (HIV positive and transplant recipients) and patients undergoing dialysis in other regions of Argentina. No differences with a control group (4.3%) were found in transplant recipients (5.8%; Pisano et al., 2017), while a higher seroprevalence of antibodies to HEV (7.3%) was found in HIV-positive patients (Debes et al., 2016) and patients undergoing hemodialysis (10.2%; Pisano et al., 2017) in Argentina, similar to findings in other countries. In a serological survey conducted in 433 patients attending primary care centers in the central region of Argentina, the seroprevalence for antibodies to HEV as detected with a commercial kit (HEV IgG ELISA, DIA.PRO, Italy) was 4.4% in 2011 (Martinez Wassaf et al., 2014). In the central region of Argentina, the seroprevalence of HEV in blood donors was much lower with a value of 1.81% in 1997 and later, in 2012 the seroprevalence increased to 9% (Rey et al., 1997; Munne et al., 2014). Recently, a surprisingly high HEV seroprevalence of 40.25% was reported in Brazil using an in-house ELISA, suggesting that in this region of Brazil, HEV is endemic (Pandolfi et al., 2017).

In Argentina, only one HEV ELISA kit is available imported from Italy and distributed from Buenos Aires to the entire country. This kind of monopoly is associated with higher costs, longer delays, and diminished accessibility. A way to circumvent this caveat is the development of in-house assays.

Therefore, we aimed to develop an ELISA to detect anti-HEV IgG antibodies that can be used for surveillance purposes and as a tool to gain knowledge on HEV epidemiology.

## Materials and Methods

### Recombinant Cloning of Hepatitis E Virus-3 ORF2

The viral antigen used in the development of the in-house ELISA was 66 kDa recombinant polypeptide comprising aa_112–608_ of the capsid protein of HEV-3. A pMK plasmid containing the coding sequence for ORF2 flanked by attB sites was obtained by synthesis at GeneArt Gene (TermoFisher Scientific) based on the ORF2 available sequence in GenBank BAG15899.1 (Takahashi et al., 2008) and further subcloned into pETG-A-His-N-[rfB] using an LR clonase (Gateway^®^ recombinatorial cloning) as described by Vizoso Pinto et al. (2010). Briefly, the LR reaction was set using 1 μl entry vector pMK-HEV3ORF2_aa112–608_, 1 μl destination vector pETG-A-His-N-[rfB], 1 μl LR clonase, and 2 μl extra pure water; the reaction was incubated 2 h at 37°C and transformed in *E. coli* DH10B by heat shock. After this, bacterial cells were plated onto LB agar added with ampicillin (100 μg/ml) and grown o.n. At least two colonies were selected, grown o.n. in LB added with ampicillin after which the plasmid was purified using a High Pure Plasmid Isolation Kit (Roche). Plasmids were checked by enzyme restriction with *HindIII* and *XbaI* (New England Biolabs) followed by agarose electrophoresis.

### Expression and Purification of RGS-His_5_-Tagged Hepatitis E Virus-3 ORF2

Chemically competent *E. coli* Rosetta (DE3) was transformed with pETG-A-His-N-ORF2 by heat shock and selected on LB plates supplemented with 100 μg/ml ampicillin and 17 μg/ml chloramphenicol. Several transformants were selected and kept in LB medium supplemented with 25% glycerol at −20°C. Overnight cultures were inoculated in fresh medium and grown for 2 h, after which protein expression was induced with different concentrations of IPTG (0.25, 0.5, 1.0, or 2.0 mM) during 1–5 h, or overnight, and at 30°C or 37°C. After centrifugation, bacterial pellets were resuspended with ice-cold lysis buffer (10% glycerol, 20 mM Tris-HCl, 0.5 M NaCl, 5 mM imidazole, pH 7.9, supplemented with 0.02 mg/ml DNAse, 0.1% Triton, 0.2 mM PMSF, 1 mM DTT, and 1 mg/ml lysozyme) and incubated on ice for 1 h. Cells were lysed by 3 cycles of freeze-thawing. The supernatant was separated by centrifugation and kept as the soluble fraction. Then, inclusion bodies were solubilized in a buffer containing 0.5 M NaCl, 5 mM imidazole, 20 mM Tris–HCl, and 8 M urea pH 7.9. The ORF2 protein was purified under native and denaturing conditions using NiNTA chromatography (Thermo Fisher Scientific) following the manufacturer’s instructions. Expression and purity of recombinant proteins were analyzed by SDS/PAGE followed by staining and verified by Western blotting using a mouse monoclonal anti-RGS-His antibody (Qiagen, Germany). Purified proteins were stored at −70°C. Protein concentration was determined with Bradford’s reagent (BioRad) following the manufacturer’s instructions.

### Serum Samples

To determine the cut-off value and performance of the in-house ELISA (specificity, sensitivity, ROC curve, and *κ* index), we used a panel of 197 serum samples (30 HEV IgG positive and 167 HEV IgG negative sera) obtained as follows: 24 serum samples belonged to patients with signs and symptoms of hepatitis and elevated transaminases of unknown origin, which were previously searched for total antibodies to HEV using the HEV Ab ELISA (DIA.PRO, Italy) for diagnostic purposes at CEMIC. Only two of the sera were also RT-PCR positive (LightMix^®^, Modular Hepatitis E Virus, Roche SAP), and the amplified product was sequenced and corresponded to genotype 3. Further, only five of these samples presented specific IgM antibodies to HEV as determined with the HEV IgM ELISA (DIA.PRO, Italy). Two of the samples were anti-HEV IgG positive samples from Inst. Malbran. A further, three anti-HEV IgG positive and 121 negative anti-HEV IgG samples belonging to our blood donor panel were also screened with HEV IgG (DIA.PRO) and therefore included in the characterized panel. All 197 samples were retested in duplicate using the HEV IgG (DIA.PRO) to confirm the results provided before.

### Enzyme-Linked Immunosorbent Assay In-House

To develop an assay to detect specific anti-HEV IgG antibodies, we optimized the antigen (recombinant ORF2) concentration, the dilution of serum, and the dilution of secondary antibody; we chose among three different blocking agents and tested the optimal TMB concentration. High-protein binding 96-well plates (JetBiofil^®^ and Nunc^®^) were coated with the purified antigen and diluted in carbonate buffer at different concentrations (0.1–20 μg/well). Plates were incubated overnight at 4°C. The wells were washed with PBS, added with 0.5% Tween-20 (PBST), and then blocked with 1% (v/v) gelatin (Sigma), 5% skim milk or 1% BSA and diluted in PBST. Human sera were serially diluted to find the optimal dilution, added to the plates, and incubated at 37°C for 60 min. Then, a HRP-secondary antibody (Dako) was tested at different dilutions, added to the wells, and incubated at 37°C for 60 min. The plates were washed with PBST and revealed with 0.1 mg/ml substrate (3,3′,5,5′-tetramethylbenzidine, Sigma), and the reaction was stopped with 1 M phosphoric acid. Plates were read on an ELISA reader (Allshen) at 450 nm.

### Evaluation of the In-House Enzyme-Linked Immunosorbent Assay Performance

To find the most appropriate cut-off value and to describe the test thoroughly, receiver-operated characteristic (ROC) analyses and calculated area under the curves (AUCs) were performed to achieve minimum target values for both sensitivity and specificity along with corresponding estimates and a Wilson binomial confidence interval. Agreement between the commercial and the in-house assays was assessed by pairwise comparisons using the *κ* coefficient.

To determine the detection limit of the assay, we serially diluted a WHO HEV serum standard (NIBSC 95/584) and tested the dilutions on the in-house ELISA plate.

The intra-assay variability was calculated as an average from the individual coefficients of variation (CV) from well to well of high, low, and negative anti-HEV-IgG samples within the same plate (10 replicates each). The inter-assay precision was calculated from the individual coefficients of variation (CV) from well to well of high, low, and negative anti-HEV-IgG samples from different plates. Acceptable criteria for intra- and inter-assay variability were defined as coefficient of variation (CV) <10 and <15%, respectively. Acceptable criteria for functional sensitivity were CV <20% (Pisanic et al., 2017).

### Commercial Enzyme-Linked Immunosorbent Assay

We determined the presence of anti-HEV IgG antibodies in the 197 serum samples of the panel using the only commercially available kit in Argentina (HEV IgG ELISA, DIA.PRO, Italy) according to the manufacturer’s instructions. The HEV IgG ELISA (DIA.PRO) is a qualitative test. Its microplates are coated with HEV-specific synthetic antigens encoding for conservative and immunodominant determinants derived from Mexican and Burmese virus strains. Serum is diluted 1:100 before testing. We used the cut-off value 0.3515 and calculated as A_450 nm (negative control)_ + 0.350, as suggested by the test’s leaflet.

### Seroprevalence Study

The minimal sample size necessary to determine the seroprevalence in blood donors of Tucumán was 126 serum samples, as calculated using the InfoStat software and the EpiTool considering an estimated seroprevalence of 7% (a mean value of the seroprevalence reported for other regions in Argentina) with a confidence level of 95%, a desired precision of 5%, and the sensitivity and specificity values obtained for the in-house ELISA (93.3 and 99.4%, respectively). Nevertheless, a larger number with a total of 813 blood bank serum samples collected in 2017 were included in the present study. Briefly, the median age was 35. Most of the participants (73.05%) were male, and 34.22% were between 26 and 35 years old. The protocol was approved by the Committee on Research Ethics of the SI.PRO.SA. (Sistema Provincial de Salud, Tucumán, Argentina, case file 849,709). The in-house HEV ELISA was used to determine anti-HEV IgG seroprevalence in 813 blood donors of Tucuman, Argentina.

### Statistical Analysis

The receiver operating characteristic (ROC) curve was used to assess the optimal cut-off values for interpretation of the results obtained with the in-house ELISA. Sensitivity and specificity were calculated.

The agreement between the in-house ELISA and the commercial ELISA (HEV IgG, DIA.PRO, Italy) was assessed using Cohen’s *κ* coefficient. Confidence intervals were calculated according to the binomial (Clopper-Pearson) “exact method” bases on the β distribution. We used *χ*^2^ at a 95% CI to compare differences between categorical variables. The values of *p* <0.05 were considered statistically significant. Confidence intervals were calculated according to the binomial (Clopper-Pearson) “exact method” bases on the β distribution. All analyses were conducted using EpiTools^1^.

CV% in the intra- and inter-assay variability was calculated with Excel^®^ (Windows^®^).

## Results

### Recombinatorial Cloning of Hepatitis E Virus-3 ORF2

After subcloning, the recombinant plasmids were confirmed by double restriction enzyme digestion (*HindIII* and *XbaI*) followed by electrophoresis on 1% agarose (data not shown).

### Expression and Purification of RGS-His_5_-Tagged Hepatitis E Virus-3 ORF2

We selected and worked with the best clone among several different *E. coli* Rosetta ORF2-expressing ones. Protein expression was induced optimally with 1 mM IPTG when A_595nm_ reached a value of 0.6 after shaking at 37°C for 3 h. The RGS-His_5_-tagged ORF2 protein was purified by NiNTA chromatography (Thermo Fischer Scientific) at higher yields under native conditions than under denaturing conditions, suggesting that most of the protein was present in the bacterial cytoplasm. The expression of the truncated ORF2 protein was checked by SDS-PAGE and Western blotting using an anti-RGS-His_5_ antibody and a HEV positive serum (data not shown). Under native conditions, ORF2 protein forms dimers and trimers; its yield was approximately 4.93 mg/L.

### Optimal Conditions for Developing an In-House Enzyme-Linked Immunosorbent Assay

**Figure 1:**
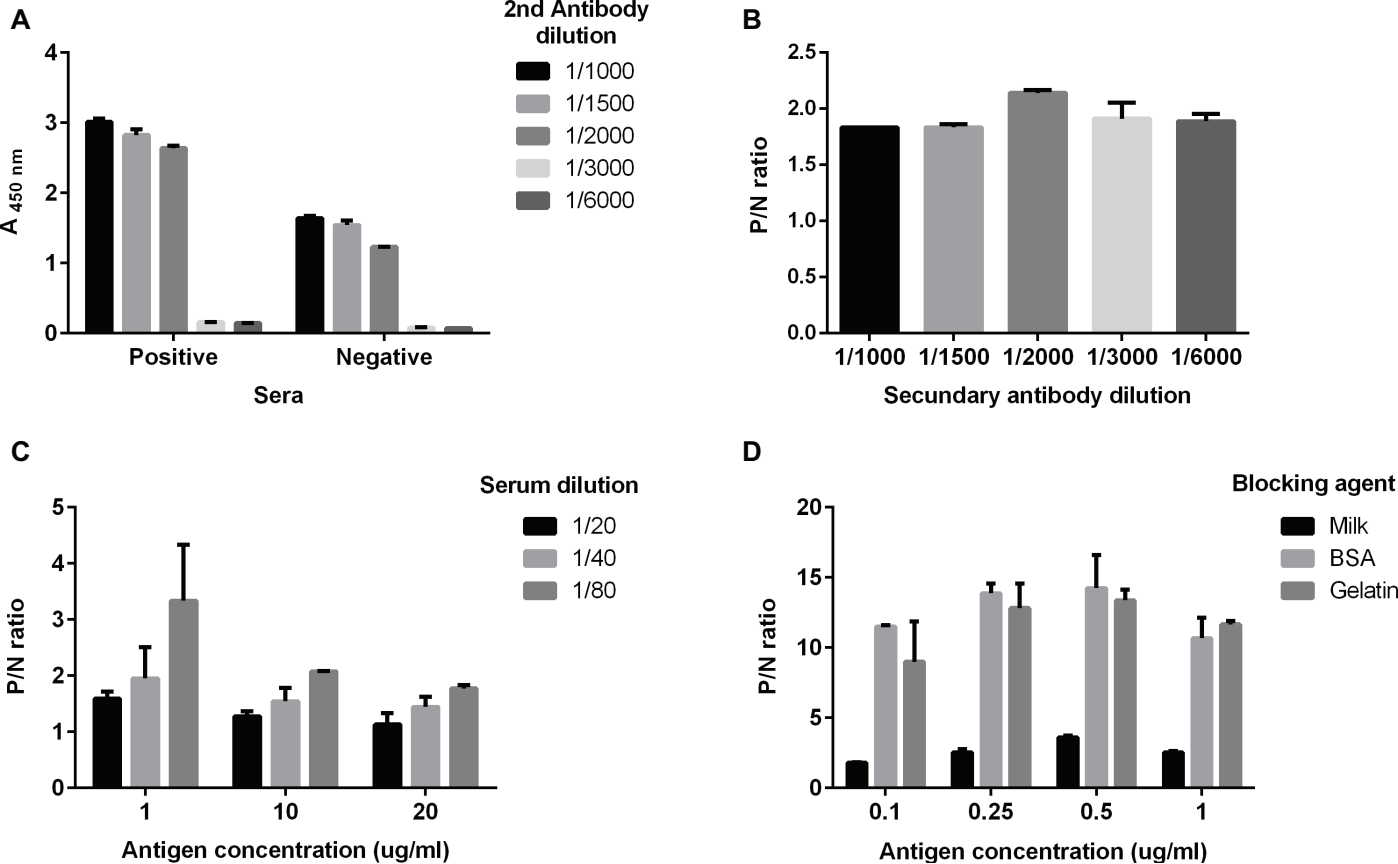
Optimal conditions for the in-house ELISA. Serum samples previously determined as positive or negative for anti-HEV IgG with the commercial kit (DIA.PRO) were used. The optimal dilutions of serum samples **(A)** and of the secondary antibody were determined **(B)** using an antigen concentration of 10 μg/ml. Serum samples positive for anti-HEV IgG or negative for anti-HEV IgG, as previously determined with the commercial assay, were used. **(C)** Optimal antigen concentration at three serum dilutions 1:20 (black balks), 1:40 (light gray balks) and 1:80 (dark gray balks) and **(D)** different blocking agents. P/N ratio in **(B–D)** is calculated as the relation A_(*λ* 450 nm)_ positive sample/A_(*λ* 450 nm)_ negative control.

The ELISA was optimized by comparing native and denatured antigens, different antigen concentrations, serum dilutions, and blocking agents, and two different plate brands. We first tried native and denatured antigens and selected the former for further development because of its better performance and yield. Then, we found the best dilution of the secondary antibody to be 10 μg/ml coating antigen with 5% milk as blocking agent (Figure 1A). Afterward, we selected the optimal serum dilution according to reactivities observed by coating plates with three different antigen concentrations (1, 10, and 20 μg/ml) plus 5% milk as blocking agent (Figure 1B). The optimal serum dilution tested was 1:80 (Figure 1C). For optimizing the relation between the positive and the negative samples (P/N), we diluted the coating antigen and tested in three blocking agents (Figure 1D); when plates were blocked with milk, negative samples exhibited a much higher background than with gelatin or BSA (*p* < 0.05). Thus, we selected a coating antigen concentration of 0.25 μg/ml, a serum dilution of 1:80, a secondary antibody dilution of 1:2000, and 1% gelatin as blocking agent. Finally, we tested two different brands of high-protein binding 96-well plates (Nunc Polysorb and JetBiofil), but they did not differ significantly from each other (data not shown). JetBiofil plates were chosen because they gave a better cost-benefit ratio.

### Relative Specificity Sensitivity of the In-House Enzyme-Linked Immunosorbent Assay

**Figure 2:**
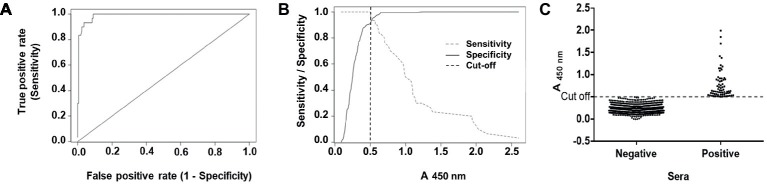
ROC curve, sensitivity and specificity graph of the in-house ELISA, and seroprevalence of anti-HEV IgG antibodies in blood donors. **(A)** ROC curve obtained with 197 characterized sera for their reactivity to HEV. **(B)** The cut-off value of the assay (A_450nm_ values = 0.498) as determined by the optimal values of the sensitivity (dashed gray line) and specificity (full black line) curves. **(C)** Prevalence of anti-HEV antibodies in 813 blood donors. Results are depicted as absorbance values at *λ* = 450 nm. Samples with A_450nm_ values > 0.498 were considered as positive using the optimal parameters determined before for the ELISA.

To calculate a proper cut-off value, receiver-operated characteristic (ROC) and two graph ROC curve analyses were performed using a panel of 197 characterized serum samples resulting in a cut-off value of A_450_ = 0.498 (Figures 2A,B). The high area under curve (AUC) value of 0.988 (95% CI: 0.976–1.0) reflects the high accuracy of the assay (Figure 2A). Further, we compared our in-house ELISA with a commercial assay (HEV IgG ELISA, DIA.PRO; Table 1). We calculated a relative sensitivity of 93.33% (95% confidence interval, 77.93–99.18%) and a specificity of 99.4% (95% confidence interval, 96.71–99.98%; Table 1) at the selected cut-off value of A_450nm_ = 0.498. The *κ* agreement test yielded a high score of 0.9402 (0.8731–1.0073), reflecting a strong to very good agreement with the DIA.PRO HEV ELISA test (Table 1).

**Table 1:** Performance of the in-house ELISA compared to the commercial ELISA (DIA.PRO) in detecting anti-HEV IgG.

		Commercial ELISA		
		Positive	Negative	Total
**In-house ELISA**	**Positive**	28	1	29
	**Negative**	2	166	168
	**Total**	30	167	**197**
**Relative sensitivity**	93.33% (95%CI, 77.93–99.18%)			
**Relative specificity**	99.4% (95%, 96.71–99.98%)			
*κ*	0.9402 (95%CI, 0.8731–1.007)			

### Detection Limit and Variability Intra- and Inter-Assay

We tested serially diluted samples of the HEV WHO standard on to the in-house ELISA plate. As a result, the lowest concentration detected by the assay was 0.25 IU/ml, while the detection limit of the DIA.PRO ELISA is 0.2 IU/ml according to reports in a comparative study done with five different assays (Norder et al., 2016). We found that the intra-assay variability of negative, low, and high positive HEV-IgG samples (10 replicates each) was 8, 4, and 4%, respectively, whereas the inter/assay variability was 6, 8, and 4%, respectively (data not shown).

### Seroprevalence of Anti-Hepatitis E Virus Immunoglobulin G in Blood Donors

We screened 813 serum samples from blood donors collected in Tucumán in 2017. We found 75 positive samples for anti-HEV IgG using the in-house ELISA (Figure 2C). Thus, the overall seroprevalence for anti-HEV IgG was of 9.23% (Clopper-Pearson exact 95% confidence interval, 7.33–11.43%). HEV seropositivity was independent of sex (*p* = 0.3015) and age (*p* = 0.8376).

## Discussion

We developed a HEV-3 ELISA based on the ORF2 recombinant protein produced in *E. coli* under native conditions. The protein ORF2 is the structural component of the capsid, the most immunogenic HEV protein, and the antigen of choice for serological diagnostics (Table 2). The C-terminal region is exposed on the surface of the capsid and harbors neutralizing epitopes, whereas the N-terminal region is hidden within the particle (Mori and Matsuura, 2011; Tang et al., 2011; Shata et al., 2012). Most of the assays reviewed in Table 2 are based on denatured ORF2 protein, which exposes the linear epitopes but not the conformational ones. Our assay differs from others (Table 2) in the nature of the antigen, which is obtained in *E. coli* under native conditions. Under the conditions used in this study, the protein forms dimers and trimers like it was previously seen by Zheng et al. (2018). Some of the assays presented in Table 2 obtained the antigen under denaturing conditions and refolded it after purification. The size of our antigen is like most of the antigens used in commercial assays. Most of the ELISAs in Table 2 use ORF2 or combinations with ORF3 from HEV-1. Only three of the assays (Mikrogen, DIA.PRO and Biomedical) include ORF2 or parts thereof belonging to HEV-1 and HEV-3. The ELISA from DIA.PRO is based on synthetic peptides covering ORF2 and ORF3 from different genotypes. We showed that the recombinant HEV-3 ORF2, under the conditions tested in this study, is enough to detect anti-HEV IgG with a high agreement with the commercial assay [*K =* 0.9402 (0.8731–1.0073)].

**Table 2:** Comparison of commercial ELISA tests for Hepatitis E and the in-house ELISA developed in this study.

Anti-HEV ELISA (IgG)	Antigen type	Genotype (GT)	Antigen size	Se^1^ (%)	Sp^2^ (%)	Reference
Wantai	Recombinant antigen ORF-2C-terminal	GT 1	211 aa	97.96	99.6	(Bendall et al., 2010; Shrestha et al., 2016; Abravanel et al., 2017)
	Recombinant antigen ORF-2C-terminal	GT 4	210 aa	93.2	97.8	(Park et al., 2012; Abravanel et al., 2013)
Axiom	Recombinant antigen ORF-2C-terminal (Burmese strain)	GT 1	n.s	95	98	(Norder et al., 2016)
Mikrogen	Recombinant antigen ORF-2C-terminal	GT 1 and 3	n.s	62	99	(Avellon et al., 2015; Norder et al., 2016)
Abbot	Recombinant antigen ORF-2 and ORF-3 (Burmese strain)	GT 1	ORF2 123 aa ORF3 full length	60	96	(Psichogiou et al., 1996; Myint et al., 2006)
Adaltis	Synthetic peptides encoded by the ORF-2 and ORF-3	GT 1 and 2	n.s	80	62.9	(Abravanel et al., 2013; Wu et al., 2014)
MP Biomedical	3 recombinant antigens, ORF-2 and ORF-3 (Burmese, Mexican strains)	GT 1 and US type 2	n.s	n.s	n.s	(Vollmer et al., 2016)
	3 recombinant proteins from ORF-2 GT2, ORF-3 GT3 and ORF-3 GT1	GT 1, 2 and 3	ORF2 GT2 42 aa ORF3 GT3 33 aa ORF3 GT1	73.3	65.3	(Schnegg et al., 2013; Wu et al., 2014)
Euroimmun	Recombinant antigen ORF-2 (USA strain)	GT 3	n.s	42	99	(Avellon et al., 2015; Norder et al., 2016; Vollmer et al., 2016)
DIA.PRO	4 synthetic peptides with conservative epitopes of ORF-2 and ORF-3	GT 1, 2, 3, and 4	n.s	98	96	(Avellon et al., 2015; Norder et al., 2016)
Genelab	Recombinant peptides ORF-2 and ORF-3C- terminal (Burmese, Mexican strains)	GT 1 and 2	n.s	50–90	93–100	(Bendall et al., 2010; Park et al., 2012)
DSI	Recombinant peptides ORF-2 and ORF-3	GT 1, 2 and 3	n.s	72	99	(Norder et al., 2016)
This study	Recombinant antigen ORF-2C- terminal	GT3	496 aa	77.93–99.18^*^	96.71–99.98^*^	

*n.s.: not specified*;

1 *Sensitivity*;

2 *Specificity*;

* *with a 95% confidence interval*.

About 2.54 μg of the purified protein was used to coat each ELISA plate. We estimate that 1972 plates can be prepared from 1 L bacterial culture (yield = 4.93 mg/L), which allows testing approximately 178,000 serum samples. Results obtained with our in-house ELISA highly agree with the ones furnished by the commercial assay DIA.PRO HEV IgG ELISA kit. We established a cut-off value of A_450nm_ = 0.498 for the in-house ELISA based on the ROC curve analysis (Figure 2B). Figure 2C depicts a common problem that several epidemiological studies have faced, including HEV seroprevalence studies, because small changes in the cut-off value may have a considerable influence on the seroprevalence rates.

In Argentina, diagnostic tests are scarcely developed and produced, in part because of regulations which prevent patenting diagnostic assays. We aim to offer an alternative to the only available assay: a test to be used in epidemiological studies with a similar sensitivity and specificity, but that is able to be produced locally at a lower cost. In order to use the in-house ELISA for diagnostic purposes, detection of anti-HEV IgM should be evaluated and validated.

The HEV seroprevalence of 9.23% (95% confidence interval, 7.33–11.43%) is within the range (4.4, 15.8%) found in central Argentina (Munne et al., 2011; Martinez Wassaf et al., 2014), and 9% found in Buenos Aires in 2012 (Munne et al., 2014) Although the IgG seropositivity does not represent active infection or carrier state but a past infection, it suggests that infection can be spread during periods of infectivity. Therefore, standardized screening provides an opportunity for public health services to address this concern. Indirect tests better suit the equipment available in routine laboratories in Latin America, where regular molecular testing is still uncommon.

Recently, 1–4% of chronic HEV infections were acquired by blood transfusion and developed persistent liver graft damage (Pawlotsky, 2014). Hewitt et al. evidenced the presence of HEV RNA in blood donations and the transmission of HEV through different blood components and described the morbidity of infected recipients (Hewitt et al., 2014). Chronic hepatitis E infection in immunocompromised patients is a serious issue, which may cause cirrhosis leading to liver failure. This underscores the new threat that HEV represents to blood transfusion safety. Some experts from industrialized countries recommend that systematic HEV testing by qRT-PCR should be implemented in blood banks to reduce the existent risk of serious complications and death (Hewitt et al., 2014). Systematic testing implies practical, economic, and logistic issues not currently solvable in Argentina. As seroprevalence of HEV changes over time, suggesting that some generations have been more exposed than others, it seems necessary to implement at least the epidemiological surveillance of HEV – with serological methods like the in-house ELISA presented here – in order to take public health decisions timely. Despite HEV circulation in northern Argentina (this study; Martinez Wassaf et al., 2014; Debes et al., 2016; Pisano et al., 2017, 2018a), differential diagnoses are barely done at the most important hospitals of Tucuman, indicating that HEV is not considered a possible etiologic agent. As HEV prevalence worldwide increases in parallel with the physicians’ awareness of the disease and the higher availability of diagnostic assays, we expect that this study will also raise the interest of health decision makers who should intercede to include indirect testing of HEV in regular diagnostic protocols.

In conclusion, the in-house ELISA developed in this work shows a very good agreement with an already licensed commercial HEV IgG ELISA (DIA.PRO, ITALY). We provide an accessible tool for studies to deepen the knowledge on HEV epidemiology in Argentina and neighboring countries. Using this in-house ELISA, we determined a seroprevalence of 9.23% (95% confidence interval, 7.33–11.43%) in northern Argentina.

## Data Availability Statement

The datasets generated for this study are available on request to the corresponding author.

## Ethics Statement

The studies involving human participants were reviewed and approved by the Committee on Research Ethics of the SI.PRO.SA (Sistema Provincial de Salud, Tucumán, Argentina, case file 849709). The patients/participants provided their written informed consent to participate in this study.

## Author Contributions

LA expressed and purified the proteins, performed all the experiments, analyzed the data, and helped draft the manuscript. MM performed some experiments. AM contributed to sample preparation and characterized sera *via* commercial ELISA and RT-PCR. GM collected serum samples, analyzed sera for blood transmitted diseases, and interviewed blood donors. FA contributed to sample preparation. AB participated in cloning and discussions of the results. MV-P conceived and designed the study, performed some experiments, analyzed the data, and wrote the manuscript with input from all the authors. All authors contributed to the final manuscript.

### Conflict of Interest

The authors declare that the research was conducted in the absence of any commercial or financial relationships that could be construed as a potential conflict of interest.

## Footnotes

^1^Sergeant, ESG, 2019. Epitools epidemiological calculators. Ausvet Pty Ltd. Available at: http://epitools.ausvet.com.au
